# Quantitative Hepatobiliary Phase Signal Analysis of HCC Nodules on Gd-EOB-DTPA-Enhanced MRI: A Potential Imaging Biomarker Associated with Disease Control After Conventional Transarterial Chemoembolization (cTACE)

**DOI:** 10.3390/jcm15176904

**Published:** 2026-09-06

**Authors:** Nicolò Brandi, Sofya Yakovleva, Maurizio Biselli, Alessandro Cucchetti, Marcello Bisulli, Anna Maria Ierardi, Cristina Mosconi, Emanuela Giampalma, Matteo Renzulli

**Affiliations:** 1 Radiology Unit, Ospedale “degli Infermi”, 48018 Faenza, Italy; 2 Department of Radiology, IRCCS Azienda Ospedaliero-Universitaria di Bologna, 40138 Bologna, Italy; sofya.yakovleva@studio.unibo.it (S.Y.); cristina.mosconi3@unibo.it (C.M.); 3 Unit of Semeiotics, Liver and Alcohol-Related Diseases, IRCCS Azienda Ospedaliero-Universitaria di Bologna, 40124 Bologna, Italy; maurizio.biselli@unibo.it; 4 Department of Medical and Surgical Sciences, University of Bologna, 40138 Bologna, Italy; emanuela.giampalma@unibo.it (E.G.); matteo.renzulli@unibo.it (M.R.); 5 Department of Medical and Surgical Sciences—DIMEC, Alma Mater Studiorum University of Bologna, 40138 Bologna, Italy; alessandro.cucchett2@unibo.it; 6 Interventional Radiology Department, AUSL Romagna Trauma Center “Maurizio Bufalini” Hospital, 47521 Cesena, Italy; marcello.bisulli@auslromagna.it; 7 Department of Health Services, Diagnostic and Interventional Radiology, Foundation IRCCS Cà Granda-Ospedale Maggiore Policlinico, 20122 Milan, Italy; amierardi@yahoo.it; 8 Radiology Unit, Morgagni-Pierantoni Hospital, AUSL Romagna, 47121 Forlì, Italy

**Keywords:** hepatocellular carcinoma, gadolinium ethoxybenzyl diethylenetriaminepentaacetic acid, magnetic resonance imaging, imaging biomarkers, transarterial chemoembolization, personalized medicine

## Abstract

**Background**: To evaluate whether quantitative signal intensity (SI) measured on the hepatobiliary phase (HBP) of gadoxetic-acid-enhanced MRI is associated with disease control rate (DCR) after conventional transarterial chemoembolization (cTACE) in patients with hepatocellular carcinoma (HCC). **Methods**: In this retrospective single-center study, 112 patients with HCC treated with cTACE between April 2018 and September 2020 and with available pre-treatment gadoxetic acid-enhanced MRI were included, with one target HCC lesion analyzed per patient. Mean HBP SI was quantitatively measured using manually drawn regions of interest (ROIs) within the target tumor, adjacent liver parenchyma, and reference tissues. Treatment response was assessed using mRECIST criteria at 1, 3, and 6 months. Logistic regression and receiver operating characteristic analyses were performed to evaluate the association between HBP SI and DCR. **Results**: Lower tumor HBP SI was associated with DCR at 6 months after cTACE (unadjusted *p* = 0.010; Bonferroni-adjusted *p* = 0.030), whereas no significant association was observed at 1 or 3 months. A tumor HBP SI cut-off value of 580 demonstrated moderate discriminatory performance for DCR (AUC = 0.714, 95% CI: 0.585–0.843; *p* = 0.008), with 69.1% sensitivity and 80.0% specificity. **Conclusions**: Lower baseline tumor HBP SI was associated with 6-month radiological disease control after cTACE in this exploratory single-center cohort. However, its moderate discriminatory performance, the absence of significant associations at earlier response assessments, and the methodological limitations of the study preclude its use as a stand-alone clinical decision-making tool. HBP SI should therefore be regarded as a potential imaging biomarker, and these findings should be considered hypothesis-generating and require validation in larger, independent cohorts.

## 1. Introduction

Hepatocellular carcinoma (HCC) represents the most prevalent primary liver malignancy, with a globally rising incidence, particularly in Western countries, driven by increasing rates of metabolic dysfunction-associated steatotic liver disease (MASLD) and chronic viral hepatitis. HCC typically develops in patients with chronic liver disease or cirrhosis and is often diagnosed at intermediate or advanced stages, when curative options such as resection or transplantation are no longer feasible [1,2,3,4,5,6].

According to the latest EASL update, transarterial chemoembolization (TACE), despite its central role in the therapeutic algorithm, is now largely regarded as a disease control strategy because of suboptimal response rates and limited likelihood of complete tumor necrosis. TACE may be applied across a wide spectrum of tumor stages, either as an alternative or in preference to surgery or systemic therapies, or as part of multidisciplinary strategies aimed at improving outcomes and achieving tumor downsizing/downstaging in individuals awaiting liver resection or transplantation [7]. Furthermore, in real-life clinical scenarios, TACE may also be considered in very early (BCLC 0) and early (BCLC A) stages in patients who are ineligible or refractory to curative therapies such as surgery or ablation [8,9,10,11]. Additionally, TACE is gaining increasing relevance in combination with immunotherapy, as emerging evidence suggests synergistic and superior oncological outcomes compared with TACE alone [12,13].

Despite its widespread use, the therapeutic benefit of TACE is highly variable. While successful outcomes rely on maximizing tumor response while minimizing injury to the surrounding hepatic parenchyma, the profound heterogeneity among HCC patients in tumor burden, liver function, and disease etiology, combined with variability in procedural factors, contributes to widely differing responses [14,15,16]. Traditional staging systems such as BCLC have been shown to inadequately stratify candidates for TACE [17,18], prompting the development of alternative scores incorporating tumor characteristics, liver function, and serological markers [19]. However, these systems often fail to integrate intrinsic tumor biological heterogeneity and have not been translated into routine clinical practice [20].

In this context, hepatobiliary contrast agents such as gadolinium ethoxybenzyl-diethylenetriaminepentaacetic acid (Gd-EOB-DTPA) have enhanced MRI-based liver assessment by enabling non-invasive characterization of hepatocellular function and liver tumor biology [21,22]. Expression of organic anion transporting polypeptide 1B3 (OATP1B3) on hepatocyte membranes gradually decreases during hepatocarcinogenesis, potentially representing its “primum movens” [23]. Since OATP1B3 mediates hepatocyte uptake of Gd-EOB-DTPA, its down-regulation results in diminished contrast enhancement and relative hypointensity of malignant nodules on HBP imaging [24]. These advances have improved early detection and characterization of dysplastic nodules and HCC and suggest that quantitative HBP signal analysis may serve as a non-invasive surrogate of tumor biology relevant to treatment response [25,26,27,28]. Recent studies have demonstrated the potential of Gd-EOB-DTPA MRI-based models to predict pathological features such as tumor differentiation, microvascular invasion, and postoperative prognosis [29,30,31,32,33]. However, these approaches have mainly focused on surgical cohorts, while evidence in the TACE setting remains limited and methodologically heterogeneous.

The aim of the present study was to evaluate the association between quantitative HBP signal analysis of HCC nodules on Gd-EOB-DTPA-enhanced MRI and radiological disease control at 6 months after conventional TACE (cTACE), while also evaluating the association at the earlier 1- and 3-month response assessments.

## 2. Materials and Methods

The present observational, retrospective, single-center study was conducted using a dataset derived from a previously approved prospective study (protocol code n. 216/2018/AOUBo), which was conducted in accordance with the Declaration of Helsinki. For the current retrospective analysis, ethical approval was waived due to the exclusive use of previously collected and published data from an already approved study, with no additional data collection or patient involvement. All analyses were performed on anonymized data, in accordance with institutional policies.

### 2.1. Patient Population and Study Design

**Figure 1:**
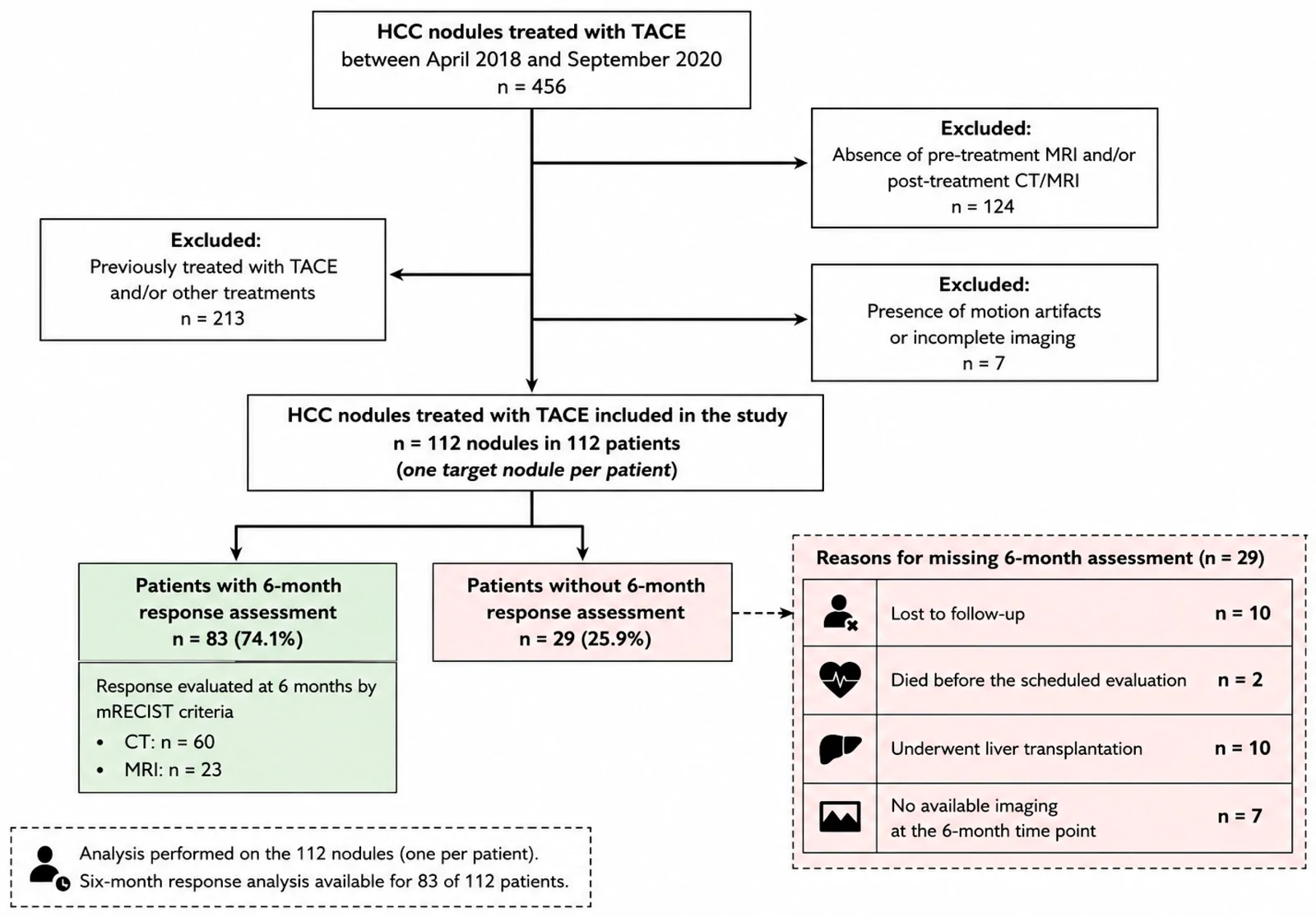
Flow diagram of patient selection in the study.

All consecutive patients with HCC who underwent cTACE between April 2018 and September 2020 at the Radiology Unit of the IRCCS Azienda Ospedaliero-Universitaria of Bologna were retrospectively evaluated. Eligibility criteria included a confirmed diagnosis of HCC based on the 2018 EASL recommendations [34], availability of pre-treatment Gd-EOB-DTPA-enhanced MRI, and at least one post-treatment contrast-enhanced CT and/or Gd-EOB-DTPA-enhanced MRI. All imaging studies were retrospectively reviewed by an experienced operator to confirm the diagnosis according to the latest EASL update (2025) [5]. Follow-up imaging at 1, 3, and/or 6 months was collected. Patients with previous TACE or other locoregional or surgical treatments were excluded to minimize potential confounding related to treatment-induced changes in tumor vascularization and imaging characteristics. Patients with incomplete data or poor-quality images due to motion artifacts were also excluded (Figure 1). Collected data included clinical variables (age, sex, liver disease etiology, presence of cirrhosis, Child–Pugh class), and tumor multiplicity and location. For each patient, a single target HCC nodule was included in the quantitative analysis, corresponding to the lesion selected for cTACE following multidisciplinary tumor board evaluation. Therefore, the study included 112 patients and 112 target HCC nodules, with one lesion-level observation per patient.

### 2.2. Imaging Techniques and Analysis

Pre- and post-treatment Gd-EOB-DTPA-enhanced MRI and post-treatment contrast-enhanced CT were performed according to international guideline standards [5]. MRI examinations were performed using the same 1.5-T MR system (Signa, GE Medical Systems, Milwaukee, WI, USA) equipped with a body phased-array multicoil and using a standardized institutional liver MRI acquisition protocol. Gd-EOB-DTPA-enhanced imaging included precontrast and dynamic contrast-enhanced T1-weighted acquisitions obtained using a three-dimensional breath-hold fast spoiled gradient-echo technique. Images were acquired with a slice thickness of 4–5 mm (effective section thickness, 2–2.5 mm). Gd-EOB-DTPA (Primovist, Bayer AG, Berlin, Germany) was administered intravenously at a dose of 0.1 mL/kg body weight and an injection rate of 1–2 mL/s, followed by a 20 mL saline flush. Hepatobiliary-phase images were acquired 20 min after contrast administration using a flip angle of 15°. The acquisition protocol was standardized throughout the study period and was consistent with previously described institutional protocols.

For quantitative analysis of pre-treatment Gd-EOB-DTPA-enhanced MRI, a region of interest (ROI) was manually drawn on the HCC nodule scheduled for cTACE, adjacent liver parenchyma, and spleen (or paraspinal muscle in splenectomized patients); care was taken to exclude visible intratumoral vessels, areas of necrosis or hemorrhage, and imaging artifacts. ROIs were placed on pre-contrast and HBP images on a single axial slice corresponding to the largest tumor diameter, including its largest possible portion. Mean, minimum, maximum signal intensity (SI), and standard deviation (SD) were recorded. Signal enhancement was quantified using an enhancement coefficient (EC), defined as the ratio of HBP SI to pre-contrast SI for each site; EC was computed on a per-lesion basis and the values reported in the tables represent the mean of individual EC values rather than the ratio of mean SI measurements.

Image analysis was independently performed by two abdominal radiologists (7 and 15 years of experience), both blinded to clinical outcomes. For each quantitative parameter, the measurements obtained by the two readers were averaged, and the resulting mean value was used for all subsequent statistical analyses. Interobserver agreement for continuous SI measurements was assessed using the intraclass correlation coefficient (ICC).

### 2.3. TACE Procedure Technique

Pre-procedural contrast-enhanced CT and/or MRI was used to assess vascular anatomy and plan the cTACE procedure, including post-processing techniques such as maximum-intensity projection (MIP).

On the day of the procedure, patients were monitored in the angiography suite with ECG, non-invasive blood pressure, and pulse oximetry. After local anesthesia, arterial access—typically via the right femoral artery—was obtained. Preliminary abdominal aortography was performed to evaluate visceral arteries and identify vascular abnormalities.

Selective catheterization of tumor-feeding hepatic arteries was performed according to lesion location. Superselective catheterization using 2.7–2.8 Fr microcatheters (Terumo Progreat or Boston Scientific Renegade) was achieved whenever possible to minimize non-tumoral liver damage. Cone-beam CT was used when feeder identification was uncertain.

Once the microcatheter was positioned, an emulsion of 10 mL iodized oil (Lipiodol, Guerbet, Villepinte, France) and 50 mg epirubicin (Farmorubicin, Pfizer, New York, NY, USA) was slowly injected, followed by embolization with gelatin sponge particles (Spongostan, Ferrosan Medical Devices A/S, Søborg, Denmark) until angiographic stasis was achieved. In cases of multiple arterial feeders, all safely accessible branches were treated. All procedures were performed by interventional radiologists with more than 15 years of experience.

### 2.4. Assessment of Treatment Response

Treatment response was evaluated on contrast-enhanced CT or MRI at 1, 3, and/or 6 months using mRECIST criteria [35]. Objective response rate (ORR) and disease control rate (DCR) were calculated; specifically, ORR was defined as CR + PR, whereas DCR was defined as CR + PR + SD. All assessments were performed by an experienced hepatobiliary radiologist.

### 2.5. Statistical Analysis

Categorical variables were reported as frequencies and percentages, and continuous variables as mean ± standard deviation (SD) or median with interquartile range (IQR), as appropriate. Variable distribution was assessed using the Skewness test. Group comparisons were performed using the χ^2^ test or Fisher’s exact test for categorical variables and the Mann–Whitney U test for continuous variables. For the longitudinal analysis of baseline HBP SI in relation to treatment response, patients achieving disease control (DCR; CR + PR + SD) were compared with those showing progressive disease (PD) separately at the 1-, 3-, and 6-month assessments. To account for multiplicity across these three time-point comparisons, Bonferroni-adjusted *p* values were additionally calculated. HBP SI values according to the individual mRECIST response categories (CR, PR, SD, and PD) were considered descriptive and were not treated as separate inferential comparisons. Both unadjusted and Bonferroni-adjusted *p* values are reported for the DCR-versus-PD comparisons. Univariate and multivariable logistic regression analyses were performed to identify MRI-derived variables associated with response to cTACE, reporting odds ratios (ORs) and 95% confidence intervals (CIs). For the exploratory multivariable analysis, four MRI-derived quantitative variables were simultaneously entered into the logistic regression model using the ENTER method: tumor mean HBP SI, tumor enhancement coefficient, tumor-to-liver HBP SI ratio, and tumor-to-spleen HBP SI ratio. These variables were selected to represent complementary quantitative aspects of tumor enhancement, including absolute HBP signal intensity, relative enhancement from the precontrast to the hepatobiliary phase, and HBP signal intensity normalized to two internal reference tissues (liver and spleen). Receiver operating characteristic (ROC) curve analysis was performed to assess the discriminatory performance of quantitative MRI parameters, and optimal cut-off values were identified using the Youden index. A *p* value < 0.05 was considered statistically significant. Given the exploratory nature of the study, analyses other than the three longitudinal DCR-versus-PD comparisons were considered exploratory and were not subjected to additional multiplicity adjustment. Analyses were conducted using SPSS version 20.0 (IBM Corp., Armonk, NY, USA).

## 3. Results

A total of 112 patients were included (86 men, 76.8%; 26 women, 23.2%), with a mean age of 72 years (range, 46–92). All HCCs diagnosed under the EASL 2018 guidelines were also confirmed using the updated 2025 guidelines [5,34]. The mean diameter of treated HCC nodules was 17.13 ± 6.71 mm (range, 8–41 mm) (Table 1). Most lesions were located in the right hepatic lobe (73.2%). The mean interval between baseline MRI and cTACE was 34.5 days. Mean hospital stay was 2.4 days (range, 2–3), including the procedure day. No periprocedural or early post-procedural complications were observed.

### 3.1. Treatment Response

Treatment response according to mRECIST was assessed on contrast-enhanced CT or MRI at 1, 3, and/or 6 months. All 112 patients (100%) underwent 1-month follow-up (80 CT, 32 MRI); follow-up at 3 and 6 months was available for 103 (92%) and 83 patients (74.1%), respectively (65 CT and 38 MRI at 3 months; 55 CT and 28 MRI at 6 months). Among the 29 patients without 6-month response assessment, 10 were lost to follow-up, two died before the scheduled evaluation, 10 underwent liver transplantation, and seven had no available imaging at the 6-month time point. Response rates are summarized in Table 2. At 6 months, 60.2% of target lesions achieved CR, 12.1% PR, 9.6% SD, and 18.1% PD, yielding an overall DCR of 81.9%.

### 3.2. Quantitative MRI Analysis

Quantitative analysis of Gd-EOB-DTPA-enhanced MRI showed a mean SI of 311.45 on pre-contrast images and 519.35 in the HBP for treated HCC nodules (EC 1.73). Surrounding hepatic parenchyma demonstrated higher values (pre-contrast SI 350.177; HBP SI 758.29; EC 2.21). The spleen (or paraspinal muscle in splenectomized patients) showed a mean pre-contrast SI of 264.33 and HBP SI of 420.21 (EC 1.64) (Table 1).

Descriptive HBP SI values according to the individual mRECIST response categories are reported in Table 3. PD lesions showed progressively higher mean HBP SI than CR, PR, or SD lesions at both 3 and 6 months. At 3 months, mean HBP SI was 581.00 for PD versus 517.76 for CR, 498.22 for PR, and 528.65 for SD; at 6 months, values were 644.13 versus 530.02, 542.00, and 485.88, respectively. Similar patterns were observed for aggregated outcomes: lesions contributing to ORR exhibited mean HBP SI of 515.18 at 3 months and 532.02 at 6 months, whereas lesions contributing to DCR showed values of 518.24 and 526.59, respectively.

Inter-reader agreement for SI measurements between the two radiologists was excellent (ICC = 0.93).

### 3.3. Association of HBP Signal Intensity with Disease Control

**Figure 2:**
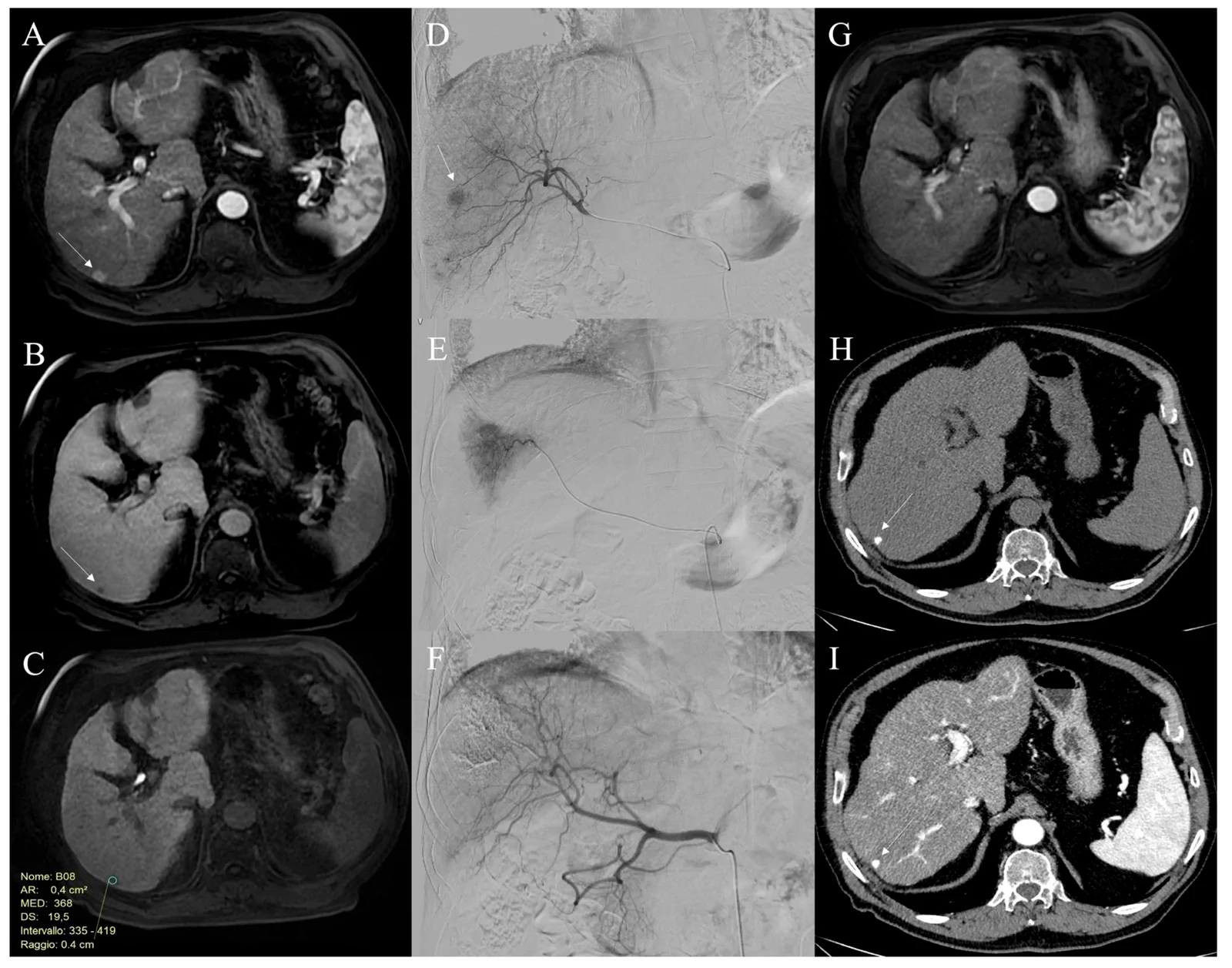
Axial contrast-enhanced MRI images acquired with a hepatospecific contrast agent showing a 10 mm HCC nodule located in segment 7, with arterial phase hyperenhancement (**A**), washout in the portal-delayed phase (**B**), and marked signal hypointensity (mean = 368) in the HBP (**C**). Angiographic images documenting the cTACE procedure with identification of the nodule (**D**), injection of chemotherapeutic agents and embolic material (**E**), and subsequent confirmation of the absence of tumor vascularization at the end of the procedure (**F**). At 6-month follow-up, an axial arterial-phase MRI image (**G**) demonstrates successful treatment, with no evidence of residual viable tumor and a CR according to mRECIST criteria. Non-contrast (**H**) and arterial-phase (**I**) axial CT images obtained at 3-month follow-up show accumulation of iodized oil (Lipiodol-Ultrafluid, LUF) at the site of the treated nodule, with no signs of residual disease.

**Figure 3:**
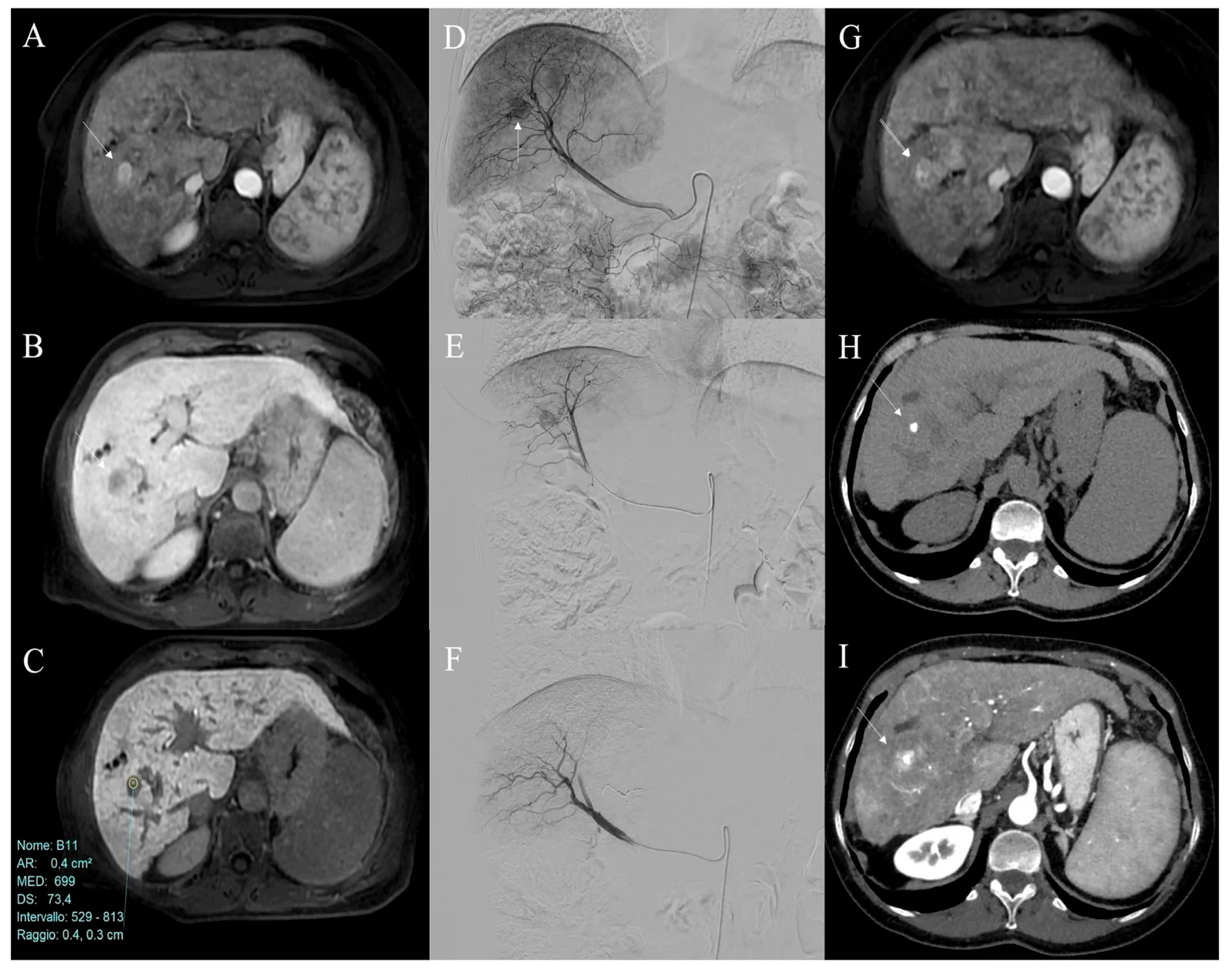
Axial contrast-enhanced MRI images acquired with a hepatospecific contrast agent showing a 14 mm HCC nodule located in segment 8, with arterial phase hyperenhancement (**A**), washout in the portal-delayed phase (**B**), and mild signal hypointensity (mean = 699) in the HBP (**C**). Angiographic images documenting the cTACE procedure with identification of the nodule (**D**), injection of chemotherapeutic agents and embolic material (**E**), and subsequent confirmation of the absence of tumor vascularization at the end of the procedure (**F**). At 6-month follow-up, an axial arterial-phase MRI image (**G**) demonstrates persistence of disease and a diameter increase (18 mm vs. 14 mm), resulting in PD according to mRECIST criteria. Non-contrast (**H**) and arterial-phase (**I**) axial CT images obtained at 6-month follow-up show accumulation of iodized oil (Lipiodol-Ultrafluid, LUF) at the site of the treated nodule, with persistence of disease at its periphery.

When HBP SI was compared across the individual mRECIST response categories (CR, PR, SD, and PD), no statistically significant differences were observed at the evaluated follow-up time points. HBP SI values according to the individual mRECIST response categories (CR, PR, SD, and PD) were descriptively evaluated at each follow-up time point (Table 3). In the comparison between DCR (CR + PR + SD) and PD, no significant association was observed at 1 month (*p* = 0.412; Bonferroni-adjusted *p* = 1.000) or 3 months (*p* = 0.158; adjusted *p* = 0.474). At 6 months, however, lower baseline tumor HBP SI was associated with disease control, with mean HBP SI values of 526.59 ± 173.37 in patients with DCR and 644.13 ± 172.49 in patients with PD (unadjusted *p* = 0.010; Bonferroni-adjusted *p* = 0.030) (Figure 2 and Figure 3).

As a sensitivity analysis addressing the use of different imaging modalities for response assessment, the association between baseline HBP SI and 6-month response was additionally evaluated after stratification by follow-up imaging modality (Appendix A, Table A1). At 6 months, 55 patients were assessed by CT and 28 by MRI. DCRs were 81.8% (45/55) and 82.1% (23/28), respectively. In the CT subgroup, baseline HBP SI was lower in patients with DCR than in those with PD (527.9 ± 189.2 vs. 647.1 ± 177.8; *p* = 0.046). The same directional difference was observed in the MRI subgroup (524.0 ± 141.2 vs. 638.2 ± 181.5; *p* = 0.093), although statistical significance was not reached in this smaller subgroup. In an additional sensitivity logistic regression analysis adjusting for follow-up imaging modality, baseline HBP SI remained associated with DCR (OR per 100-SI-unit increase, 0.70; 95% CI, 0.51–0.96; *p* = 0.028). No significant interaction between HBP SI and follow-up imaging modality was observed (*p* = 0.676).

The exploratory multivariable logistic regression model included HBP SI, enhancement coefficient, tumor-to-liver HBP SI ratio, and tumor-to-spleen HBP SI ratio (Table 4). Tumor HBP SI was associated with 6-month disease control (OR = 0.995, 95% CI: 0.991–0.999; *p* = 0.020), whereas enhancement coefficient (OR = 2.341, 95% CI: 0.382–14.352; *p* = 0.358), tumor-to-liver HBP SI ratio (OR = 0.132, 95% CI: 0.005–3.303; *p* = 0.218), and tumor-to-spleen HBP SI ratio (OR = 3.673, 95% CI: 0.159–84.764; *p* = 0.417) were not significantly associated with disease control. The overall multivariable model did not reach statistical significance (*p* = 0.056).

**Figure 4:**
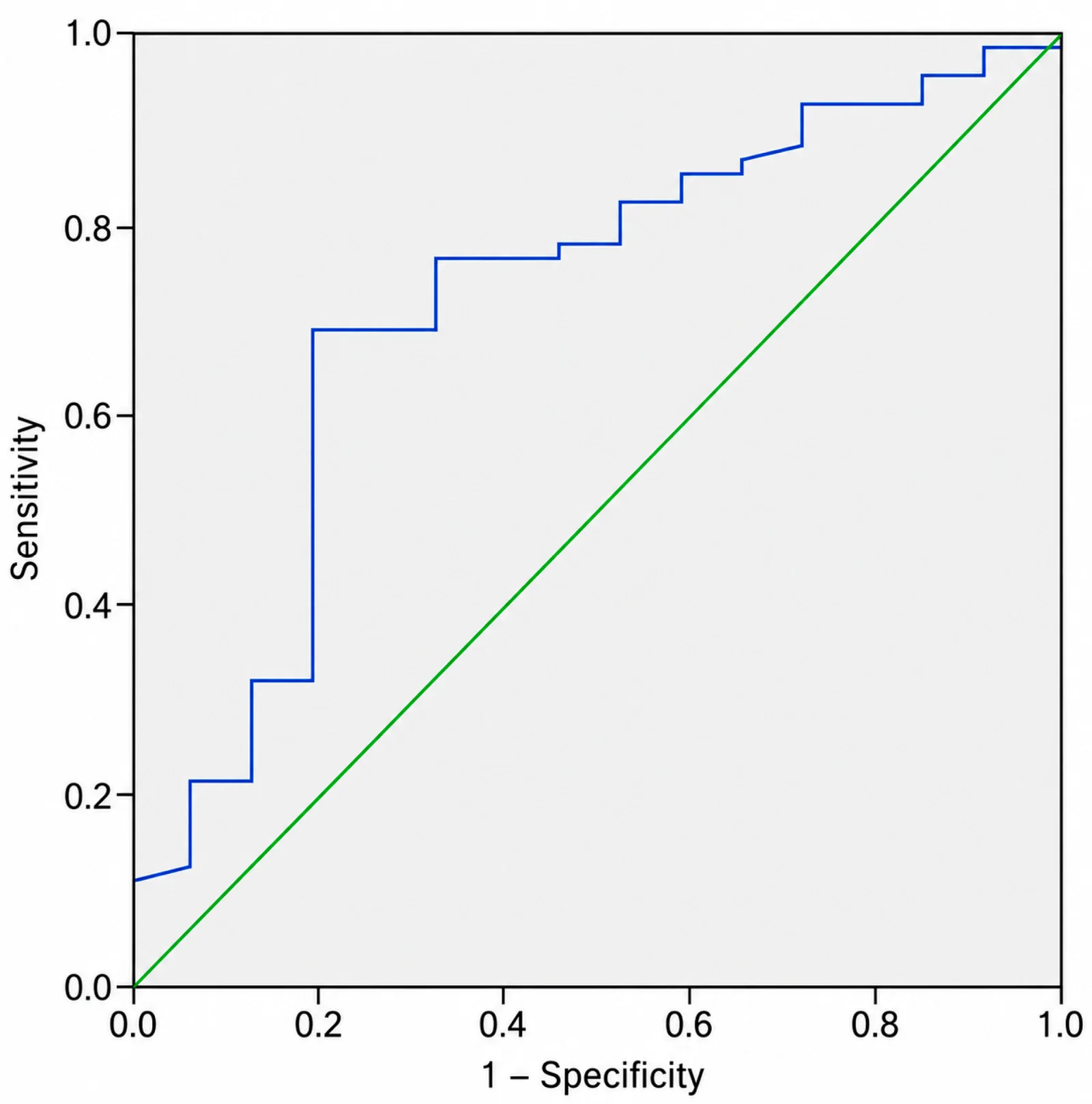
ROC curve illustrating sensitivity (*y*-axis) versus 1 − specificity (*x*-axis) for baseline tumor HBP SI in discriminating disease control from progressive disease at 6 months. The optimal cut-off value of 580.00 was identified using the Youden index (AUC = 0.714, 95% CI: 0.585–0.843; *p* = 0.008).

ROC analysis of baseline tumor HBP SI for discrimination between disease control and PD at 6 months yielded an AUC of 0.714 (95% CI: 0.585–0.843; *p* = 0.008). An HBP SI threshold of 580 showed a sensitivity of 69.1% and a specificity of 80.0% for discriminating between the two outcome groups (Figure 4). Given the exploratory nature of the analysis, this threshold should be regarded as a cohort-specific value rather than a clinically validated cut-off.

## 4. Discussion

The present study suggests that quantitative assessment of HBP SI on Gd-EOB-DTPA-enhanced MRI may provide a simple non-invasive imaging parameter associated with disease control after cTACE in patients with HCC. Lower baseline tumor HBP SI was associated with DCR at 6 months. The discriminatory performance of HBP SI should, however, be interpreted cautiously. The observed AUC of 0.714 (95% CI, 0.585–0.843) indicates only moderate discrimination and is insufficient to support the use of HBP SI as a stand-alone clinical decision-making tool. Moreover, the relatively wide confidence interval highlights the uncertainty surrounding the estimated discriminatory performance in this relatively small cohort. Accordingly, the HBP SI threshold of 580 should be regarded as an exploratory, cohort-derived cut-off rather than as a clinically validated threshold. External validation in larger independent cohorts is required before its potential clinical applicability can be established.

This association is biologically plausible, as HBP enhancement reflects OATP1B3 expression, which mediates hepatocellular uptake of Gd-EOB-DTPA and is progressively downregulated during hepatocarcinogenesis [36,37,38]. Although direct correlations between HBP enhancement and histological grade in humans are limited, preclinical and clinical evidence indicates that lower HBP SI may be associated with tumor dedifferentiation, aggressive behavior, and worse prognosis, supporting HBP hypointensity as a potential indirect imaging marker of tumor biology [30,31,39,40,41,42,43,44]. However, histological grade and OATP1B3 expression were not directly assessed in our cohort. Therefore, the biological interpretation of the observed association remains hypothetical.

Tumor dedifferentiation has also been associated with progressive changes in vascular supply, from mixed portal–arterial inflow in earlier stages toward predominantly arterial perfusion during HCC progression [36,45,46,47]. Well-differentiated HCCs, which appear less hypointense on HBP imaging, appear relatively hypovascular and may therefore be less susceptible to TACE, which relies on arterial hypervascularization; moreover, the persistence of a partial portal contribution may further contribute to resistance to treatment-induced ischemia [48,49,50]. Conversely, biologically more advanced HCCs are characterized by a greater arterial supply and thus are generally more responsive to intra-arterial therapies, being more susceptible to abrupt interruption of blood flow and exposed to higher intratumoral concentrations of chemotherapeutic agents [49,51,52]. In line with this hypothesis, previous studies have reported poorer response rates and higher recurrence after TACE in well-differentiated, relatively hypovascular lesions [53,54,55,56,57,58].

In later stages, in addition to almost completely losing OATP1B3 expression, poorly differentiated HCCs also exhibit a progressive reduction in arterial supply due to rapid cell proliferation and the subsequent increased interstitial compression [59,60], ultimately losing the typical arterial hypervascularization that underlies the effectiveness of TACE. Although this biological framework may appear counterintuitive, as lower HBP SI has been associated with more advanced tumor dedifferentiation, changes in portal and arterial blood supply during hepatocarcinogenesis may provide a potential mechanistic explanation for the observed association. In particular, this apparent paradox may be related to the differential contribution of portal venous supply to HCC nodules. While well-differentiated nodules often retain portal inflow—potentially contributing to resistance after arterial embolization [61,62]—poorly differentiated HCCs typically lose portal vascularization and may depend predominantly on a limited residual arterial microcirculation [51,63]. Occlusion of this remaining arterial supply may therefore induce severe ischemic injury and effective tumor necrosis [61]. Nonetheless, neither histological differentiation nor quantitative portal and arterial tumor perfusion was directly assessed in the present study. Therefore, this proposed mechanism cannot be demonstrated by our data and should be considered hypothesis-generating.

Only a limited number of studies have evaluated the role of gadoxetic-acid-enhanced MRI in predicting response after locoregional therapies in HCC. Lee et al. [64] demonstrated that visually assessed heterogeneous HBP SI was an independent predictor of non-complete response after DEB-TACE, supporting the concept that intratumoral heterogeneity reflects unfavorable tumor biology. However, their qualitative approach is subject to interpretation. Similarly, Minamiguchi et al. [65] reported that quantitative measures of HBP signal heterogeneity, expressed as the coefficient of variation, were associated with poorer overall survival after TACE in intermediate-stage HCC, reinforcing its prognostic relevance beyond subjective visual assessment. Nevertheless, the study was limited by a relatively small sample size and methodological heterogeneity, including sequential treatments of the same nodule and variable protocols (bland TAE in tumors > 6 cm and DEB-TACE in patients > 80 years), which may have acted as confounding factors and limited generalizability. Other studies have described better response and lower early recurrence in HCC nodules with paradoxical or increased HBP uptake, likely reflecting better differentiation and preserved OATP expression [58,66]. However, hyperintense HBP lesions were not included in the present study since they are relatively rare (<10%) [24].

This study has several limitations. First, its retrospective, single-center design may limit generalizability; however, the monocentric setting ensured homogeneous imaging protocols, standardized cTACE techniques, and consistent follow-up assessment, thereby reducing procedural and technical variability. The inclusion of treatment-naïve patients and the target-lesion-based approach increased cohort homogeneity but may limit the generalizability of our findings to previously treated patients and to the full complexity of multifocal HCC encountered in clinical practice. Although consecutive patient screening was used to reduce discretionary selection, selection bias cannot be excluded because eligibility required adequate pre-treatment Gd-EOB-DTPA-enhanced MRI and available post-treatment imaging. Moreover, given the limited number of PD events at 6 months relative to the number of variables included, and because the multivariable model was restricted to MRI-derived parameters without comprehensive adjustment for relevant clinical and tumor-related confounders, the adjusted estimates may be susceptible to model instability and should not be interpreted as demonstrating an independent predictive effect of HBP SI. In addition, multiple MRI-derived parameters and response categorizations were explored, which increases the possibility of type I error. To address multiplicity in the longitudinal analysis of HBP SI, a Bonferroni correction was applied across the three DCR-versus-PD comparisons performed at 1, 3, and 6 months; the association observed at 6 months remained statistically significant after adjustment (adjusted *p* = 0.030). Nevertheless, given the exploratory nature of the study and the additional analyses performed, the findings should be considered hypothesis-generating and require confirmation in a larger independent cohort.

Additionally, SI measurements were obtained using a two-dimensional ROI on a single axial slice rather than volumetric segmentation, a deliberate choice aimed at maximizing simplicity, time efficiency, and clinical translatability. Although this approach provides a readily applicable quantitative measurement, it cannot fully capture spatial intratumoral heterogeneity and may be affected by sampling variability. In this context, radiomics and artificial intelligence (AI)-based imaging approaches may provide a more comprehensive quantitative characterization of imaging heterogeneity and are increasingly being investigated for lesion characterization, treatment-response assessment, and personalized risk stratification [67,68,69]. Recent developments also include automated segmentation methods supporting quantitative imaging workflows [70], delta-radiomics approaches for assessing longitudinal treatment-related changes in imaging features [71], and the integration of radiomics-derived information into interventional radiology workflows [72]. Compared with these more complex approaches, conventional HBP SI measurement has the potential advantage of simplicity and clinical accessibility, as it can be obtained from routine Gd-EOB-DTPA-enhanced MRI using standard ROI measurements without dedicated radiomics or machine-learning pipelines. Conversely, this simplicity comes at the cost of a less comprehensive representation of intratumoral heterogeneity. Future studies should therefore investigate whether combining HBP SI with volumetric radiomics or AI-derived features provides incremental value for predicting response to cTACE.

A further relevant limitation concerns the use of an absolute HBP SI cut-off value, which may be influenced by scanner-related factors, acquisition parameters, and vendor-specific characteristics, potentially affecting reproducibility across different institutions. Accordingly, given both its moderate discriminatory performance and its potential dependence on scanner- and acquisition-related factors, the proposed threshold should be regarded as an exploratory, cohort-derived value rather than a clinically validated or universally applicable cut-off. Although inter-reader reproducibility of SI measurements was excellent (ICC = 0.93), the reproducibility and generalizability of absolute SI values and of the proposed cut-off across different scanners, vendors, field strengths, and acquisition protocols remain uncertain and require external validation. Although signal normalization to reference tissues may theoretically improve inter-scanner comparability, normalized enhancement metrics did not provide additional predictive value in our cohort, possibly because absolute tumor HBP uptake more directly reflects intrinsic tumor biology, whereas normalization may introduce variability related to background liver function or systemic factors. Future multicenter studies should determine whether relative or normalized HBP metrics can enhance generalizability while preserving biological relevance.

Additionally, tumor vascularity and the accuracy of feeder vessel selection are well-known determinants of cTACE efficacy. These procedural and angiographic factors were not quantitatively analyzed in the present study and therefore represent a limitation. However, all procedures were performed by highly experienced interventional radiologists using a standardized superselective technique, minimizing variability related to technical execution. Importantly, the aim of the study was not to model procedural success, but to investigate whether pre-treatment hepatobiliary phase signal intensity reflects intrinsic tumor biological features that may contribute to treatment response alongside procedural and angiographic factors.

Furthermore, 6-month response assessment was available for 83 of the 112 patients initially included. Among the 29 non-evaluable patients, the reasons for missing assessment included loss to follow-up, death, liver transplantation, and unavailable imaging. Because some of these reasons may have been related to the patients’ clinical course, missingness cannot be assumed to have occurred at random, and consequent selection/attrition bias may have influenced the observed 6-month association. In addition, follow-up response assessment was not performed using a uniform imaging modality, with most 6-month evaluations performed by CT rather than MRI (55 vs. 28 examinations). In the post-cTACE setting, iodized-oil deposition may complicate CT-based assessment of residual viable enhancing tumor, potentially introducing modality-related variability in mRECIST classification. In the modality-stratified analysis, however, lower baseline HBP SI was observed in patients achieving DCR compared with those with PD in both the CT and MRI subgroups, although the difference did not reach statistical significance in the smaller MRI subgroup. Furthermore, adjustment for follow-up imaging modality did not abolish the overall association between HBP SI and 6-month disease control, and no significant HBP SI-by-modality interaction was observed. Nevertheless, these subgroup findings should be interpreted cautiously because of the limited sample size, particularly the small number of PD events in the MRI subgroup. Therefore, modality-related variability cannot be definitively excluded, and prospective studies using a standardized follow-up imaging modality remain warranted.

Furthermore, clinically relevant longitudinal outcomes such as overall survival, progression-free survival, and time to progression were not assessed in the present study. Therefore, the observed association between baseline HBP SI and radiological disease control cannot be directly translated into a survival benefit or broader long-term prognostic significance. Finally, the association between baseline HBP SI and treatment outcome was observed at 6 months but not at the earlier 1- and 3-month assessments. The 6-month DCR-versus-PD association remained statistically significant after Bonferroni correction for the three longitudinal time-point comparisons (adjusted *p* = 0.030). Accordingly, HBP SI should not be interpreted as a predictor of early radiological response. Rather, the 6-month finding may suggest a potential association with the persistence of disease control over time. Importantly, no additional anticancer treatments were administered before the 6-month assessment, reducing the potential for treatment-related confounding. However, this time-dependent association remains exploratory and requires prospective validation. Early post-treatment assessments in the present cohort may have had limited statistical power, particularly because of the small number of PD events. Moreover, early imaging after TACE may overestimate treatment efficacy, as TACE may initially induce tumor growth arrest rather than immediate tumor cell death, allowing residual viable cells to resume proliferation over time [73,74]. Treatment-induced hypoxia may also promote angiogenesis and tumor progression through VEGF upregulation, potentially contributing to delayed treatment failure [75]. Consistently, previous studies have suggested that mRECIST may correlate more reliably with pathological response at later rather than earlier assessments [76,77]. Accordingly, the potential clinical value of baseline HBP SI, if confirmed, would relate to the identification of patients more likely to maintain radiological disease control over time rather than to the prediction of immediate post-cTACE response.

Despite these limitations, quantitative HBP SI measurement represents a simple and rapid attractive imaging tool that does not require advanced software or specialized expertise. Similar to what has been proposed for other hepatic malignancies [78], current guidelines already acknowledge a selective role for Gd-EOB-DTPA-enhanced MRI in specific clinical scenarios. If independently validated, baseline HBP SI could potentially provide complementary imaging information for the multidisciplinary assessment of patients considered for cTACE, rather than serving as a stand-alone criterion for treatment selection. Notably, in patients in whom HCC diagnosis is established on contrast-enhanced CT, this information could potentially be obtained by integrating the diagnostic workup with a limited MRI protocol focused exclusively on the HBP, thereby reducing examination time, scanner workload, and overall costs. Compared with radiomics-based approaches and complex AI-driven models, a single quantitative HBP SI value offers greater interpretability and feasibility, acting as a bridge between advanced imaging biomarkers and everyday clinical practice, without the need for dedicated software and trained personnel [79]. Future studies should focus on integrating quantitative HBP SI with histological and molecular biomarkers, including direct assessment of OATP1B3 expression and β-catenin pathway activation.

## 5. Conclusions

In conclusion, in this exploratory single-center study, lower baseline tumor HBP SI was associated with radiological disease control at 6 months after cTACE, whereas no significant association was observed at the earlier 1- and 3-month assessments. The 6-month association remained significant after correction for multiple time-point comparisons. However, given the moderate discriminatory performance and the methodological limitations of the study, HBP SI should be regarded as a potential, hypothesis-generating imaging biomarker rather than a stand-alone clinical decision-making tool. Prospective multicenter studies are needed to validate these results across different imaging platforms and to determine whether HBP SI may provide complementary information for future treatment stratification alongside established clinical and imaging parameters.

## Figures and Tables

**Table 1:** Descriptive clinical and demographic characteristics of the study population, including the assessment of quantitative signal features of HCC nodules treated with TACE.

	Total (*n* = 112)
**Age** [mean (±SD)]	72.7 (±10.8)
**Sex** [n (%)]	
Male	86 (76.8%)
Female	26 (23.2%)
**Etiology of liver disease** [n (%)]	
Viral	65 (58.0%)
Non-viral	47 (42.0%)
**Child–Pugh class** [n (%)]	
A	94 (83.9%)
B or C	18 (16.1%)
**BCLC** [n (%)]	
**A**	50 (44.6%)
**B**	62 (55.4%)
**AFP** [ng/mL, median (IQR)]	5.85 (3.4–18.8)
**Maximum Diameter** [mean (±SD)]	17.13 (±6.71)
**Location** [n (%)]	
Right lobe	82 (73.2%)
Left lobe	29 (25.9%)
Caudate lobe	1 (0.9%)
**Quantitative signal features of HCC nodules** [mean (±SD)]	
Pre-contrast SI	311.45 (±98.81)
HBP SI	519.35 (±181.14)
EC	1.73 (±0.54)
**Quantitative signal features of the liver parenchyma** [mean (±SD)]	
Pre-contrast SI	350.177 (±95.70)
HBP SI	758.29 (±246.88)
EC	2.21 (±0.57)
**Quantitative signal features of the spleen** [mean (±SD)]	
Pre-contrast SI	264.33 (±97.59)
HBP SI	420.21 (±126.44)
EC	1.64 (±0.37)

**Table 2:** Treatment response according to mRECIST at 1, 3, and/or 6 months.

Treatment Response According to mRECIST [n(%)]			
	1-Month Follow-Up (n = 112)	3-Month Follow-Up (n = 103)	6-Month Follow-Up (n = 83)
**CR**	69 (61.6%)	59 (57.3%)	50 (60.2%)
**PR**	15 (13.4%)	9 (8.7%)	10 (12.1%)
**SD**	27 (24.1%)	20 (19.4%)	8 (9.6%)
**PD**	1 (0.9%)	15 (14.6%)	15 (18.1%)

**Table 3:** HBP SI of HCC nodules stratified by treatment response according to mRECIST criteria and by aggregated response endpoints (ORR and DCR) at 1, 3, and 6 months.

HBP SI of HCC Nodules Based on Treatment Response [Mean (±SD)]			
	1-Month Follow-Up (n = 112)	3-Month Follow-Up (n = 103)	6-Month Follow-Up (n = 83)
**CR**	525.54 (±204.04)	517.76 (±205.11)	530.02 (±192.87)
**PR**	492.40 (±115.25)	498.22 (±161.43)	542.00 (±116.00)
**SD**	523.11 (±151.37)	528.65 (±144.78)	485.88 (±87.63)
**PD**	395.00	581.00 (±147.42)	644.13 (±172.49)
**ORR** (CR + PR)	519.62 (±191.08)	515.18 (±198.93)	532.02 (±181.57)
**DCR** (CR + PR + SD)	520.47 (±181.57)	518.24 (±187.31)	526.59 (±173.37)
**DCR vs. PD** (*p* value)	0.412	0.158	**0.010**
Bonferroni-adjusted *p* value	1.000	0.474	**0.030**

Values for individual response categories and aggregated endpoints are descriptive. Inferential testing compared lesions achieving DCR (CR + PR + SD) with those showing PD at each follow-up assessment. Bonferroni correction was applied across the three time-point comparisons (1, 3, and 6 months). The DCR-versus-PD comparison was significant only at 6 months (unadjusted *p* = 0.010; Bonferroni-adjusted *p* = 0.030). Bold values indicate statistical significance (*p* < 0.05).

**Table 4:** Exploratory multivariable logistic regression analysis for disease control at 6 months after cTACE.

Variable	B	SE	Wald	*p*-Value	OR (Exp[B])	95% CI for OR
**Tumor Mean HBP SI**	−0.005	0.002	5.393	**0.020**	**0.995**	**0.991–0.999**
**Tumor EC**	0.851	0.925	0.845	0.358	2.341	0.382–14.352
**Tumor-to-liver HBP SI ratio**	−2.022	1.642	1.518	0.218	0.132	0.005–3.303
**Tumor-to-spleen HBP SI ratio**	1.301	1.601	0.660	0.417	3.673	0.159–84.764

B: regression coefficient; SE: standard error. All four MRI-derived variables were simultaneously entered into the model using the ENTER method. Bold values indicate statistical significance (*p* < 0.05).

## Data Availability

The datasets generated or analyzed during the study are available from the corresponding author on reasonable request.

## Abbreviations

The following abbreviations are used in this manuscript:

HCC	Hepatocellular Carcinoma
MASLD	Metabolic dysfunction-Associated Steatotic Liver Disease
EASL	European Association for the Study of the Liver
TACE	Transarterial Chemoembolization
BCLC	Barcelona Clinic Liver Cancer
HBP	Hepatobiliary Phase
Gd-EOB-DTPA	Gadolinium Ethoxybenzyl-diethylenetriaminepentaacetic Acid
MRI	Magnetic Resonance Imaging
OATP1B3	Organic Anion Transporting Polypeptide 1B3
cTACE	Conventional Transarterial Chemoembolization
ROI	Region of Interest
SD	Standard Deviation
SI	Signal Intensity
EC	Enhancement Coefficient
MIP	Maximum Intensity Projection
ORR	Objective Response Rate
DCR	Disease Control Rate
OR	Odds Ratio
CI	Confidence Interval
ROC	Receiver Operating Characteristic
AFP	Alpha Fetoprotein
B	Regression Coefficient
SE	Standard Error
AUC	Area Under the Curve
DEB-TACE	Drug-Eluting Beads Transarterial Chemoembolization
TAE	Transarterial Embolization
VEGF	Vascular Endothelial Growth Factor
AI	Artificial Intelligence

## Appendix A

**Table A1:** The 6-month radiological response and baseline tumor HBP signal intensity according to follow-up imaging modality.

6-Month Assessment	CT (n = 55)	MRI (n = 28)
DCR, n (%)	45 (81.8)	23 (82.1)
PD, n (%)	10 (18.2)	5 (17.9)
HBP SI in DCR, mean ± SD	527.9 ± 189.2	524.0 ± 141.2
HBP SI in PD, mean ± SD	647.1 ± 177.8	638.2 ± 181.5
DCR vs. PD, *p* value	0.046	0.093

*p*-values refer to the comparison of baseline HBP SI between DCR and PD within each imaging-modality subgroup using the Mann–Whitney U test.

## References

[1] Bray F., Laversanne M., Sung H., Ferlay J., Siegel R.L., Soerjomataram I., Jemal A. (2024). Global cancer statistics 2022: GLOBOCAN estimates of incidence and mortality worldwide for 36 cancers in 185 countries. CA Cancer J. Clin..

[2] Dasgupta P., Henshaw C., Youlden D.R., Clark P.J., Aitken J.F., Baade P.D. (2020). Global trends in incidence rates of primary adult liver cancers: A systematic review and meta-analysis. Front. Oncol..

[3] Renzulli M., Brandi N., Pecorelli A., Pastore L.V., Granito A., Martinese G., Tovoli F., Simonetti M., Dajti E., Colecchia A. (2022). Segmental distribution of hepatocellular carcinoma in cirrhotic livers. Diagnostics.

[4] Guo Z., Wu D., Mao R., Yao Z., Wu Q., Lv W. (2025). Global burden of MAFLD, MAFLD-related cirrhosis and MASH-related liver cancer from 1990 to 2021. Sci. Rep..

[5] European Association for the Study of the Liver (2025). EASL clinical practice guidelines on the management of hepatocellular carcinoma. J. Hepatol..

[6] Singal A.G., Llovet J.M., Yarchoan M., Mehta N., Heimbach J.K., Dawson L.A., Jou J.H., Kulik L.M., Agopian V.G., Marrero J.A. (2023). AASLD practice guidance on prevention, diagnosis, and treatment of hepatocellular carcinoma. Hepatology.

[7] Pommergaard H.C., Rostved A.A., Adam R., Thygesen L.C., Salizzoni M., Bravo M.A.G., Cherqui D., De Simone P., Boudjema K., Mazzaferro V. (2018). Locoregional treatments before liver transplantation for hepatocellular carcinoma: A study from the European Liver Transplant Registry. Transpl. Int..

[8] Xue T.C., Xie X.Y., Zhang L., Yin X., Zhang B.H., Ren Z.G. (2013). Transarterial chemoembolization for hepatocellular carcinoma with portal vein tumor thrombus: A meta-analysis. BMC Gastroenterol..

[9] Deng J., Liao Z., Gao J. (2023). Efficacy of transarterial chemoembolization combined with tyrosine kinase inhibitors for hepatocellular carcinoma patients with portal vein tumor thrombus: A systematic review and meta-analysis. Curr. Oncol..

[10] Guo L., Wei X., Feng S., Zhai J., Guo W., Shi J., Lau W.Y., Meng Y., Cheng S. (2022). Radiotherapy prior to or after transcatheter arterial chemoembolization for the treatment of hepatocellular carcinoma with portal vein tumor thrombus: A randomized controlled trial. Hepatol. Int..

[11] Park J.W., Chen M., Colombo M., Roberts L.R., Schwartz M., Chen P.J., Kudo M., Johnson P., Wagner S., Orsini L.S. (2015). Global patterns of hepatocellular carcinoma management from diagnosis to death: The BRIDGE study. Liver Int..

[12] Brandi N., Renzulli M. (2023). The synergistic effect of interventional locoregional treatments and immunotherapy for the treatment of hepatocellular carcinoma. Int. J. Mol. Sci..

[13] Kudo M., Ren Z., Guo Y., Han G., Lin H., Zheng J., Ogasawara S., Kim J.H., Zhao H., Li C. (2025). Transarterial chemoembolisation combined with lenvatinib plus pembrolizumab versus dual placebo for unresectable, non-metastatic hepatocellular carcinoma (LEAP-012): A multicentre, randomised, double-blind, phase 3 study. Lancet.

[14] Giannini E.G., Moscatelli A., Pellegatta G., Vitale A., Farinati F., Ciccarese F., Piscaglia F., Rapaccini G.L., Di Marco M., Caturelli E. (2016). Application of the intermediate-stage sub-classification to patients with untreated hepatocellular carcinoma. Am. J. Gastroenterol..

[15] Raoul J.L., Sangro B., Forner A., Mazzaferro V., Piscaglia F., Bolondi L., Lencioni R. (2011). Evolving strategies for the management of intermediate-stage hepatocellular carcinoma: Available evidence and expert opinion on the use of transarterial chemoembolization. Cancer Treat. Rev..

[16] Renzulli M., Peta G., Vasuri F., Marasco G., Caretti D., Bartalena L., Spinelli D., Giampalma E., D’eRrico A., Golfieri R. (2021). Standardization of conventional chemoembolization for hepatocellular carcinoma. Ann. Hepatol..

[17] Cho Y.K., Chung J.W., Kim J.K., Ahn Y.S., Kim M.Y., Park Y.O., Kim W.T., Byun J.H. (2008). Comparison of 7 staging systems for patients with hepatocellular carcinoma undergoing transarterial chemoembolization. Cancer.

[18] Terzi E., Piscaglia F., Forlani L., Mosconi C., Renzulli M., Bolondi L., Golfieri R. (2014). TACE performed in patients with a single nodule of hepatocellular carcinoma. BMC Cancer.

[19] Granito A., Facciorusso A., Sacco R., Bartalena L., Mosconi C., Cea U.V., Cappelli A., Antonino M., Modestino F., Brandi N. (2021). TRANS-TACE: Prognostic role of the transient hypertransaminasemia after conventional chemoembolization for hepatocellular carcinoma. J. Pers. Med..

[20] Müller L., Stoehr F., Mähringer-Kunz A., Hahn F., Weinmann A., Kloeckner R. (2021). Current strategies to identify patients that will benefit from TACE treatment and future directions: A practical step-by-step guide. J. Hepatocell. Carcinoma.

[21] Candita G., Rossi S., Cwiklinska K., Fanni S.C., Cioni D., Lencioni R., Neri E. (2023). Imaging diagnosis of hepatocellular carcinoma: A state-of-the-art review. Diagnostics.

[22] Renzulli M., Brandi N., Argalia G., Brocchi S., Farolfi A., Fanti S., Golfieri R. (2022). Morphological, dynamic and functional characteristics of liver pseudolesions and benign lesions. Radiol. Med..

[23] Brandi N., Renzulli M. (2024). Liver lesions at risk of transformation into hepatocellular carcinoma in cirrhotic patients: Hepatobiliary phase hypointense nodules without arterial phase hyperenhancement. J. Clin. Transl. Hepatol..

[24] Narita M., Hatano E., Arizono S., Miyagawa-Hayashino A., Isoda H., Kitamura K., Taura K., Yasuchika K., Nitta T., Ikai I. (2009). Expression of OATP1B3 determines uptake of Gd-EOB-DTPA in hepatocellular carcinoma. J. Gastroenterol..

[25] Ichikawa T., Sano K., Morisaka H. (2014). Diagnosis of pathologically early HCC with EOB-MRI: Experiences and current consensus. Liver Cancer.

[26] Hanna R.F., Miloushev V.Z., Tang A., Finklestone L.A., Brejt S.Z., Sandhu R.S., Santillan C.S., Wolfson T., Gamst A., Sirlin C.B. (2016). Comparative 13-year meta-analysis of the sensitivity and positive predictive value of ultrasound, CT, and MRI for detecting hepatocellular carcinoma. Abdom. Radiol..

[27] Duncan J.K., Ma N., Vreugdenburg T.D., Cameron A.L., Maddern G. (2017). Gadoxetic acid-enhanced MRI for the characterization of hepatocellular carcinoma: A systematic review and meta-analysis. J. Magn. Reson. Imaging.

[28] Renzulli M., Pecorelli A., Brandi N., Brocchi S., Tovoli F., Granito A., Carrafiello G., Ierardi A.M., Golfieri R. (2022). The feasibility of liver biopsy for undefined nodules in patients under surveillance for hepatocellular carcinoma: Is biopsy really a useful tool?. J. Clin. Med..

[29] Erra P., Puglia M., Ragozzino A., Maurea S., Liuzzi R., Sabino G., Barbuto L., Cuocolo A., Imbriaco M. (2015). Appearance of hepatocellular carcinoma on gadoxetic acid-enhanced hepatobiliary phase MR imaging: A systematic review. Radiol. Med..

[30] Kogita S., Imai Y., Okada M., Kim T., Onishi H., Takamura M., Fukuda K., Igura T., Sawai Y., Morimoto O. (2010). Gd-EOB-DTPA-enhanced magnetic resonance images of hepatocellular carcinoma: Correlation with histological grading and portal blood flow. Eur. Radiol..

[31] Tong H.F., Liang H.B., Mo Z.K., Guan T.P., Yang J., Fang C.H. (2017). Quantitative analysis of gadoxetic acid-enhanced magnetic resonance imaging predicts histological grade of hepatocellular carcinoma. Clin. Imaging.

[32] Zhang K., He K., Zhang L., Li W.C., Xie S.S., Cui Y.Z., Lin L.-Y., Shen Z.-W., Su X.-M., Zhang H.-M. (2025). Gadoxetic acid-enhanced MRI scoring model to predict pathologic features of hepatocellular carcinoma. Radiology.

[33] Pei J., Wang L., Li H. (2025). Development of a better nomogram for prediction of preoperative microvascular invasion and postoperative prognosis in hepatocellular carcinoma patients: A comparison study. J. Comput. Assist. Tomogr..

[34] European Association for the Study of the Liver (2018). EASL Clinical Practice Guidelines: Management of hepatocellular carcinoma. J. Hepatol..

[35] Lencioni R., Llovet J.M. (2010). Modified RECIST (mRECIST) assessment for hepatocellular carcinoma. Semin. Liver Dis..

[36] Kitao A., Matsui O., Yoneda N., Kozaka K., Shinmura R., Koda W., Kobayashi S., Gabata T., Zen Y., Yamashita T. (2011). The uptake transporter OATP8 expression decreases during multistep hepatocarcinogenesis: Correlation with gadoxetic acid-enhanced MR imaging. Eur. Radiol..

[37] Vasuri F., Golfieri R., Fiorentino M., Capizzi E., Renzulli M., Pinna A.D., Grigioni W.F., D’errico-Grigioni A. (2011). OATP1B1/1B3 expression in hepatocellular carcinomas treated with orthotopic liver transplantation. Virchows Arch..

[38] Renzulli M., Braccischi L., D’Errico A., Pecorelli A., Brandi N., Golfieri R., Albertini E., Vasuri F. (2022). State-of-the-art review on the correlations between pathological and magnetic resonance features of cirrhotic nodules. Histol. Histopathol..

[39] Ni Y., Marchal G., Yu J., Mühler A., Lukito G., Baert A.L. (1994). Prolonged positive contrast enhancement with Gd-EOB-DTPA in experimental liver tumors: Potential value in tissue characterization. J. Magn. Reson. Imaging.

[40] Vogl T.J., Kümmel S., Hammerstingl R., Schellenbeck M., Schumacher G., Balzer T., Schwarz W., Müller P.K., Bechstein W.O., Mack M.G. (1996). Liver tumors: Comparison of MR imaging with Gd-EOB-DTPA and Gd-DTPA. Radiology.

[41] Marchal G., Zhang X., Ni Y., Van Hecke P., Yu J., Baert A.L. (1993). Comparison between Gd-DTPA, Gd-EOB-DTPA, and Mn-DPDP in induced HCC in rats: A correlation study of MR imaging, microangiography, and histology. Magn. Reson. Imaging.

[42] Fowler K.J., Burgoyne A., Fraum T.J., Hosseini M., Ichikawa S., Kim S., Kitao A., Lee J.M., Paradis V., Taouli B. (2021). Pathologic, molecular, and prognostic radiologic features of hepatocellular carcinoma. Radiographics.

[43] Braga F.A., Torres U.S., Iared W., D’Ippolito G. (2023). Does hypointense HCC in the hepatobiliary phase at gadoxetate-enhanced MRI predict recurrence after surgery? A systematic review and meta-analysis. Acad. Radiol..

[44] Choi S.Y., Kim S.H., Park C.K., Min J.H., Lee J.E., Choi Y.H., Lee B.R. (2018). Imaging features of gadoxetic acid-enhanced and diffusion-weighted MR imaging for identifying cytokeratin 19-positive hepatocellular carcinoma: A retrospective observational study. Radiology.

[45] Hayashi M., Matsui O., Ueda K., Kawamori Y., Gabata T., Kadoya M. (2002). Progression to hypervascular hepatocellular carcinoma: Correlation with intranodular blood supply evaluated with CT during intraarterial injection of contrast material. Radiology.

[46] Kobayashi S., Matsui O., Gabata T., Koda W., Minami T., Ryu Y., Kozaka K., Kitao A. (2012). Intranodular signal intensity analysis of hypovascular high-risk borderline lesions of HCC that illustrate multistep hepatocarcinogenesis within the nodule on Gd-EOB-DTPA-enhanced MRI. Eur. J. Radiol..

[47] Asayama Y., Yoshimitsu K., Nishihara Y., Irie H., Aishima S., Taketomi A., Honda H. (2008). Arterial blood supply of hepatocellular carcinoma and histologic grading: Radiologic-pathologic correlation. AJR Am. J. Roentgenol..

[48] Huppertz A., Haraida S., Kraus A., Zech C.J., Scheidler J., Breuer J., Helmberger T.K., Reiser M.F. (2005). Enhancement of focal liver lesions at gadoxetic acid-enhanced MR imaging: Correlation with histopathologic findings and spiral CT-initial observations. Radiology.

[49] Maesaka K., Sakamori R., Yamada R., Tahata Y., Urabe A., Shigekawa M., Kodama T., Hikita H., Tatsumi T., Takehara T. (2020). Hypovascular hepatic nodules as a predictive factor for transcatheter arterial chemoembolization refractoriness in hepatocellular carcinoma. Hepatol. Res..

[50] Yoon S.H., Lee J.M., So Y.H., Hong S.H., Kim S.J., Han J.K., Choi B.I. (2009). Multiphasic MDCT enhancement pattern of hepatocellular carcinoma smaller than 3 cm in diameter: Tumor size and cellular differentiation. AJR Am. J. Roentgenol..

[51] Choi J.Y., Lee J.M., Sirlin C.B. (2014). CT and MR imaging diagnosis and staging of hepatocellular carcinoma: Part I. Development, growth, and spread: Key pathologic and imaging aspects. Radiology.

[52] Park H.J., Choi B.I., Lee E.S., Park S.B., Lee J.B. (2017). How to differentiate borderline hepatic nodules in hepatocarcinogenesis: Emphasis on imaging diagnosis. Liver Cancer.

[53] Yamashita Y., Takahashi M., Koga Y., Saito R., Nanakawa S., Hatanaka Y., Sato N., Nakashima K., Urata J., Yoshizumi K. (1991). Prognostic factors in the treatment of hepatocellular carcinoma with transcatheter arterial embolization and arterial infusion. Cancer.

[54] Katyal S., Oliver J.H., Peterson M.S., Chang P.J., Baron R.L., Carr B.I. (2000). Prognostic significance of arterial phase CT for prediction of response to transcatheter arterial chemoembolization in unresectable hepatocellular carcinoma: A retrospective analysis. AJR Am. J. Roentgenol..

[55] Chung G.E., Lee J.H., Yoon J.H., Myung S.J., Lee K., Jang J.J., Lee J.M., Kim S.-H., Suh K.-S., Kim Y.J. (2012). Prognostic implications of tumor vascularity and its relationship to cytokeratin 19 expression in patients with hepatocellular carcinoma. Abdom. Imaging.

[56] Hu H.T., Kim J.H., Lee L.S., Kim K.A., Ko G.Y., Yoon H.K., Sung K.-B., Gwon D.I., Shin J.H., Song H.-Y. (2011). Chemoembolization for hepatocellular carcinoma: Multivariate analysis of predicting factors for tumor response and survival in a 362-patient cohort. J. Vasc. Interv. Radiol..

[57] Cheung T.T., Poon R.T., Jenkins C.R., Chu F.S., Chok K.S., Chan A.C., Tsang S.H.Y., Dai W.C., Yau T.C.C., Chan S.C. (2014). Survival analysis of high-intensity focused ultrasound therapy vs. transarterial chemoembolization for unresectable hepatocellular carcinomas. Liver Int..

[58] Kim J.W., Lee C.H., Park Y.S., Seo T.S., Song M.G., Kim J.H., Kim K.A., Park C.M. (2017). The value of paradoxical uptake of hepatocellular carcinoma on the hepatobiliary phase of gadoxetic acid-enhanced liver magnetic resonance imaging for the prediction of lipiodol uptake after transcatheter arterial chemoembolization. Eur. J. Radiol..

[59] El-Assal O.N., Yamanoi A., Soda Y., Yamaguchi M., Igarashi M., Yamamoto A., Nabika T., Nagasue N. (1998). Clinical significance of microvessel density and vascular endothelial growth factor expression in hepatocellular carcinoma and surrounding liver: Possible involvement of vascular endothelial growth factor in the angiogenesis of cirrhotic liver. Hepatology.

[60] Asayama Y., Yoshimitsu K., Irie H., Nishihara Y., Aishima S., Tajima T., Hirakawa M., Ishigami K., Kakihara D., Taketomi A. (2007). Poorly versus moderately differentiated hepatocellular carcinoma: Vascularity assessment by computed tomographic hepatic angiography in correlation with histologically counted number of unpaired arteries. J. Comput. Assist. Tomogr..

[61] Goseki N., Nosaka T., Endo M., Koike M. (1995). Nourishment of hepatocellular carcinoma cells through the portal blood flow with and without transcatheter arterial embolization. Cancer.

[62] Mukund A., Kumar N., Srivastava A., Baby A. (2025). Transarterial chemoembolization: A consistent and continuously evolving therapy for hepatocellular carcinoma. J. Clin. Exp. Hepatol..

[63] Yoneda N., Matsui O., Kobayashi S., Kitao A., Kozaka K., Inoue D., Yoshida K., Minami T., Koda W., Gabata T. (2019). Current status of imaging biomarkers predicting the biological nature of hepatocellular carcinoma. Jpn. J. Radiol..

[64] Lee J.Y., Lee B.C., Kim H.O., Heo S.H., Shin S.S., Jeong Y.Y. (2021). Liver MRI and clinical findings to predict response after drug-eluting bead transarterial chemoembolization in hepatocellular carcinoma. Sci. Rep..

[65] Minamiguchi K., Nishiofuku H., Saito N., Sato T., Taiji R., Matsumoto T., Maeda S., Chanoki Y., Tachiiri T., Kunichika H. (2023). Quantitative analysis of signal heterogeneity in the hepatobiliary phase of pretreatment gadoxetic acid-enhanced MRI as a prognostic imaging biomarker in transarterial chemoembolization for intermediate-stage hepatocellular carcinoma. Cancers.

[66] Ishimaru H., Nakashima K., Sakugawa T., Sakamoto A., Matsuoka Y., Ashizawa K., Uetani M. (2014). Local recurrence after chemoembolization of hepatocellular carcinoma: Uptake of gadoxetic acid as a new prognostic factor. AJR Am. J. Roentgenol..

[67] Haghshomar M., Rodrigues D., Kalyan A., Velichko Y., Borhani A. (2024). Leveraging radiomics and AI for precision diagnosis and prognostication of liver malignancies. Front. Oncol..

[68] Haghshomar M., Kierans A.S., Kulik L., Miller F.H., Borhani A.A. (2025). Imaging-based diagnosis of hepatocellular carcinoma: Liver Imaging Reporting and Data System and beyond. Br. J. Radiol..

[69] Benfante V., Salvaggio G., Ali M., Cutaia G., Salvaggio L., Salerno S., Busè G., Tulone G., Pavan N., Di Raimondo D., Foresti G.L., Fusiello A., Hancock E. (2024). Grading and Staging of Bladder Tumors Using Radiomics Analysis in Magnetic Resonance Imaging. Image Analysis and Processing—ICIAP 2023 Workshops.

[70] Pavone A.M., Benfante V., Stefano A., Mamone G., Milazzo M., Di Pizza A., Parenti R., Maruzzelli L., Miraglia R., Comelli A., Mazzeo P.L., Frontoni E., Sclaroff S., Distante C. (2022). Automatic liver segmentation in pre-TIPS cirrhotic patients: A preliminary step for radiomics studies. Image Analysis and Processing—ICIAP 2022 Workshops.

[71] Nardone V., Patanè V., Marinelli L., D’Ambrosio L., Del Tufo S., De Chiara M., Brunese M.C., Rubini D., Grassi R., Russo A. (2025). Delta-radiomics biomarker in colorectal cancer liver metastases treated with cetuximab plus avelumab (CAVE Trial). Diagnostics.

[72] Marinelli L., Patanè V., Monti R., Tarantino N.M., Nardone V., Reginelli A., Cappabianca S. (2026). From passive sampling to precision intervention: A standardized radiomics-driven workflow for molecularly adequate lung biopsy. Cardiovasc. Interv. Radiol..

[73] Sergio A., Cristofori C., Cardin R., Pivetta G., Ragazzi R., Baldan A., Girardi L., Cillo U., Burra P., Giacomin A. (2008). Transcatheter arterial chemoembolization (TACE) in hepatocellular carcinoma (HCC): The role of angiogenesis and invasiveness. Am. J. Gastroenterol..

[74] Kim J.H., Sinn D.H., Shin S.W., Cho S.K., Kang W., Gwak G.Y., Paik Y.-H., Lee J.H., Koh K.C., Paik S.W. (2017). The role of scheduled second TACE in early-stage hepatocellular carcinoma with complete response to initial TACE. Clin. Mol. Hepatol..

[75] Liang B., Zheng C.S., Feng G.S., Wu H.P., Wang Y., Zhao H., Qian J., Liang H.-M. (2010). Correlation of hypoxia-inducible factor 1alpha with angiogenesis in liver tumors after transcatheter arterial embolization in an animal model. Cardiovasc. Interv. Radiol..

[76] Hyun D., Shin S.W., Cho S.K., Park K.B., Park H.S., Choo S.W., Do Y.S., Choo I.-W., Shin J.-W., Lim S.-J. (2015). Efficacy of RECIST and mRECIST criteria as prognostic factors in patients undergoing repeated iodized oil chemoembolization of intermediate-stage hepatocellular carcinoma. Acta Radiol..

[77] Kim J.W., Seong J., Park M.S., Kim K.S., Park Y.N., Han K.H., Keum K.C., Lee I.J. (2016). Radiological-pathological correlation study of hepatocellular carcinoma undergoing local chemoradiotherapy and surgery. J. Gastroenterol. Hepatol..

[78] Renzulli M., Clemente A., Ierardi A.M., Pettinari I., Tovoli F., Brocchi S., Peta G., Cappabianca S., Carrafiello G., Golfieri R. (2020). Imaging of colorectal liver metastases: New developments and pending issues. Cancers.

[79] Linton-Reid K., Chen M., Martell M.B., Posma J.M., Aboagye E.O. (2025). Radiomics in clinical radiology: Advances, challenges, and future directions. Clin. Radiol..

